# Factors Affecting Early Cholecystectomy for Acute Cholecystitis in Older People—A Population-Based Study

**DOI:** 10.1007/s00268-023-06968-9

**Published:** 2023-05-03

**Authors:** Jakob K. Köstenbauer, Robert C. Gandy, Jacqueline Close, Lara Harvey

**Affiliations:** 1 Department of Gastrointestinal Surgery, Royal North Shore Hospital, Sydney, NSW 2065 Australia; 2 Prince of Wales Hospital and Community Health Services, Sydney, NSW Australia; 3 Falls, Balance and Injury Research Centre, Neuroscience Research Australia, Sydney, NSW Australia; 4 Prince of Wales Clinical School, University of New South Wales, Sydney, NSW Australia; 5 University of New South Wales, Sydney, NSW Australia

## Abstract

**Objectives:**

Acute cholecystitis is one of the most common surgical presentations in Australia and increases with age. Guidelines recommend early laparoscopic cholecystectomy (within 7 days), as it results in shorter length of stay, reduced costs and readmission rates. Despite this, there is a perception that early cholecystectomy may result in higher morbidity and conversion to open surgery in older patients. Our objective is to report the proportion of early versus delayed cholecystectomy in older patients in New South Wales (NSW), Australia, and to compare health outcomes and factors influencing variation.

**Design:**

This is a retrospective population-based cohort study of all cholecystectomies for primary acute cholecystitis in NSW residents aged >50, between 2009 and 2019. The primary outcome was the proportion of early versus delayed cholecystectomy. We used multilevel multivariable logistic regression analyses adjusted for age, sex, comorbidities, insurance status, socio-economic status and hospital characteristics.

**Results:**

A high rate (85%) of the 47,478 cholecystectomies in older patients were performed within 7 days of admission. Delayed surgery was associated with increasing age and comorbidity, male sex, Medicare-only insurance and surgery in low- or medium-volume centres. Early surgery was associated with shorter overall length of stay, fewer readmissions, less conversion to open surgery and lower bile duct injury rates.

**Conclusion:**

A high proportion of adults with cholecystitis are undergoing early cholecystectomy in NSW. Our results support the efficacy of early cholecystectomy in older patients and identify potentially modifiable factors relevant to health care professionals and policymakers.

## Introduction

Acute cholecystitis is one of the most common emergency surgical presentations globally [1]. In 2016–2017 there were approximately 8800 hospitalisations for acute cholecystitis in patients aged ≥ 50 years in New South Wales (NSW), Australia [2]. Age has been identified as a strong risk factor for the development of gallstones, and older patients are more likely to present later with more severe disease [3].

Cholecystectomy remains the gold standard of treatment and can be performed early during acute admission or as a delayed elective procedure. Early cholecystectomy is defined as either open or laparoscopic cholecystectomy within seven days of presentation with acute cholecystitis. Delayed cholecystectomy is typically performed six to twelve weeks after symptom onset [1]. Early laparoscopic cholecystectomy is widely accepted as safe, widely available and cost-efficient compared to delayed surgery [4]. Global guidelines now recommend early laparoscopic cholecystectomy, regardless of age or disease severity [3].

Despite this, there remains a belief that the technical challenges of laparoscopic cholecystectomy for acute cholecystitis necessitate delaying surgery in high-risk cases—an ill-defined label often applied to older patients [3]. It is, therefore, not uncommon for older patients to be managed conservatively in the acute setting, followed by delayed cholecystectomy. This practice is at odds with current literature where several trials have demonstrated a significantly higher morbidity, recurrence rate and cost associated with delayed cholecystectomy [5–7].

Significant variation in the rate of early cholecystectomy has been observed internationally with rates from the UK, USA, Japan and Canada ranging from 50 to 84%.[8, 9]. The current rate of early cholecystectomy for acute cholecystitis in Australia is unknown [4]. The primary aim of this study is to report the rate of early versus delayed cholecystectomy in the older population of NSW. Secondly, we aim to identify patient- and system-level factors associated with delayed cholecystectomy and, finally, to report any difference in outcomes including mortality, length of stay (LOS), hospital readmission rates, major bile duct injury and conversion from laparoscopic to open procedure.

## Methods

### Data source

Data were drawn from the Surgical Care of Older People (SCOPE) cohort, which comprises all individuals aged ≥ 50 years admitted under a surgical speciality to all public and private NSW hospitals, between 1 January 2007 and 30 July 2019. The SCOPE dataset includes hospital records from the Admitted Patient Data Collection (APDC) and mortality records from the Register for Births, Deaths and Marriages (RBDM) probabilistically linked by the Centre for Health Record Linkage. The APDC is administered by the NSW Health Department. Data are submitted weekly by public hospitals or monthly by private facilities. Data submission is a requirement by law, and the department implements a range of data quality controls to ensure input accuracy [10]. Ethics approval was obtained from the NSW Population and Health Services Research Ethics Committee (2018HRE0201).

### Identification of study cohort

Patients aged  ≥ 50 years, admitted between 1 July 2008 and 30 June 2018 with a primary diagnosis of acute cholecystitis (ICD-10-AM: K80.0, K80.1, K80.4, K81.0, K81.8, K81.9), were included in the study cohort, allowing for an 18-month washout period from 1 January 2007 and a 1-year look forward period to 30 July 2019. The washout period was used to ensure only patients with an index (first) episode of acute cholecystitis were included, and the look forward period allowed identification of surgery or readmission within 1 year of the index admission. Patients undergoing cholecystostomy (ACHI: 30375-05; 30375-26; 30440-01) were excluded as these cases likely had severe cholecystitis with comorbidities rendering them unfit for surgery, or acute cholecystitis was not their primary diagnosis.

### Outcome measures

The primary outcome was early versus late cholecystectomy. Cholecystectomy was identified using Australian Classification of Health Interventions (ACHI) codes: 30443-00, 30445-00, 30446-00, 30448-00, 30449-00, 30454-01, 30455-00. Early surgery was defined as cholecystectomy within 7 days of admission and delayed surgery as cholecystectomy between 7 and 365 days after admission. Secondary outcomes were: 30-day and 90-day all-cause mortality, LOS, 28-day emergency readmission, readmission for gallstone-related disease within 1 year, conversion to open rates and incidence of major bile duct injury within 1 year of cholecystectomy (ACHI: 30472-00, 30472-01, 30470-00).

### Covariates

Patient-level characteristics included age, sex, Charlson Comorbidity Index (CCI) calculated using the Quan ICD-10-AM coding algorithm [11] with a one-year look-back period, insurance status at time of admission, socio-economic status using the Australian Socio-Economic Index for Areas–Index of Relative Socio-economic Disadvantage (SEIFA-IRSD) [12, 13] and concurrent diagnosis of pancreatitis or cholangitis on index admission. Hospital-level factors were: hospital status (classified as public tertiary, public non-tertiary, or private) and surgical volume defined as mean annual volume of cholecystectomies performed. LOS was calculated as the total days for index hospitalisation plus (where relevant) subsequent days for delayed cholecystectomy. To capture unplanned emergencies, we also calculated the duration of index hospitalisation plus all subsequent inpatient days with any gallstone-related disease. Outliers in LOS (>3 SD mean) were excluded as they likely represented atypical care or coding errors [14].

### Statistical analysis

Descriptive analyses were used to show the demographic characteristics of the cohort and assess differences between patients who underwent early versus delayed surgery. Multilevel multivariable logistic regression models were fitted to assess the influence of patient and hospital factors on timing of surgery. By adjusting for hospital factors (surgical volume and referral status) and controlling covariates at both patient and hospital levels, the model accounted for the non-independence of individuals undergoing surgery within the same hospital. Adjusted odds ratios (ORs) with 95% confidence intervals (CIs) were calculated. Akaike information criterion (AIC) was used to assess model fit. Analyses were conducted using SAS Enterprise Guide v7.1 (SAS Institute Inc, Cary NC).

## Results

### Demographics

The study cohort comprised 47,478 patients who underwent cholecystectomy for acute cholecystitis across 134 hospitals. Of these, 40,187 (85%) were performed within seven days of admission (Table 1). On further analysis, 74% were performed within 24 h, 81% within 72 h and 82% at 96 h.

**Table 1:** Characteristics of patients undergoing early or delayed cholecystectomy for acute cholecystitis

	Early Surgery *n* = 40 187 (84.6%)	Delayed Surgery *n* = 7 291 (15.4%)	*P* value
Individual characteristics			
Age, mean ± SD (range)	66 ± 10.2 (50–108)	68 ± 10.7 (50–97)	<.0001
Age group (years)			
50–64	20317 (50.6)	2979 (40.9)	<.0001
65–79	15652 (39.0)	3068 (42.1)	
80–84	2701 (6.7)	758 (10.4)	
85 +	1517 (3.8)	486 (6.7)	
Male sex	16511 (41.1)	3894 (53.4)	< .0001
Categorised weighted CCI score^^^			
0	30835 (76.7)	5211 (71.5)	<.0001
1	5198 (12.9)	1031 (14.1)	
2	1947 (4.8)	492 (6.6)	
3 +	2207 (5.5)	557 (7.6)	
Insurance status (private)^#^	12 707 (32.7)	2372 (34.3)	0.0126
Socio-economic status, quintiles*			
1—Least disadvantaged	11568 (29.3)	2138 (29.8)	0.0008
2	9971 (25.3)	1960 (27.3)	
3	7119 (18.1)	1239 (17.3)	
4	5086 (12.9)	854 (11.9)	
5—Most disadvantaged	5687 (14.4)	987 (13.8)	
Remoteness of residence^¥^			
Major city	29966 (68.4)	4268 (59.5)	<.0001
Inner regional	9147 (23.2)	2085 (29.0)	
Outer regional	3104 (7.9)	770 (10.7)	
Remote/very remote	227 (0.6)	56 (0.8)	
ICU admission	1483 (3.7)	200 (2.7)	< 0001
Pancreatitis	756 (1.9)	262 (3.6)	<.0001
Cholangitis	305 (0.8)	164 (2.3)	< .0001
Hospital characteristics			
Hospital status			
Public tertiary referral	12326 (30.7)	1863 (25.5)	<.0001
Public non-tertiary	19338 (48.1)	3298 (45.2)	
Private	8523 (21.2)	2130 (29.1)	
Annual volume cholecystectomies			
High (top quintile)	8944 (22.3)	1097 (15.1)	<.0001
Medium	23656 (58.9)	4351 (59.7)	
Low (bottom quintile)	7587 (18.9)	1843 (25.3)	

^#^ Missing data for *n* = 1,747 (3.7%) of individuals

^*^SES = Socio-economic status, by SEIFA score. Missing data for *n* = 869 (1.8%)

^¥^Accessibility/Remoteness Index of Australia (ARIA +) based on statistical local area of residence. Missing data for 885 people (1.8%)

^^^CCI = Weighted Charlson Comorbidity Index score (0 = no comorbidity)

*ICU* Intensive care unit

Table 1 shows the demographic characteristics of those undergoing early versus delayed cholecystectomy. Increasing age, male sex and higher level of comorbidity were associated with delayed surgery. Of the cholecystectomies performed in private hospitals, 8523 (80%) were completed within seven days, compared to 31,664 (79%) in public hospitals (*p* < 0.001).

There were 224 (0.5%) post-operative deaths at 90 days, 105 of which occurred within 30 days with no significant difference between groups (Table 2). The total number of major bile duct injuries was low in both groups at 73 (0.18%) in the early group versus 24 (0.33%) in the delayed surgery group (*p* = 0.0103). LOS was consistently greater for those undergoing delayed surgery (mean 8 (5.5SD) vs. 3.2 (3.7SD) days, *p* < 0.001), and all-cause emergency readmissions almost doubled in the delayed group (1002 (13.7%) vs. 2506 (6.2%)).

**Table 2:** Outcomes of patients undergoing early or delayed cholecystectomy for acute cholecystitis

	Early surgery *n* = 40 187 (84.6%)	Delayed surgery *n* = 7 291 (15.4%)	*P* value
Length of stay (days, median [IQR])			
Index + readmission for surgery	2 [1–4]	7 [4–11]	< .0001
Index + all gallstone-related readmissions within 1 year	2 [1–4]	7 [4–11]	< .0001
Length of stay (days, mean (SD))			
Index + readmission for surgery	3.1 (3.5)	7.9 (5.3)	< .0001
Index + all gallstone-related readmissions within 1 year	3.2 (3.7)	8.0 (5.5)	< .0001
28-day emergency all-cause readmissions	2506 (6.2)	1002 (13.7)	< .0001
Surgical technique			
Laparoscopic	37 724 (93.9)	6 448 (88.4)	< .0001
Open	1604 (4.0)	557 (7.6)	
Converted to open	859 (2.1)	286 (3.9)	
Major bile duct injury within 1 year	73 (0.18)	24 (0.33)	0.0103
Mortality^#^			
30-day	92 (0.2)	10 (0.1)	0.1196
9 0-day	179 (0.5)	38 (0.5)	0.3770

^#^2 individuals with inconsistent dates of death excluded

The multivariate multilevel logistic regression model confirmed that increasing age and comorbidity were significantly and independently associated with delayed surgery (Table 3). Surgery in a low-volume hospital was strongly associated with delayed surgery (OR 2.02, 95%CI 1.22–3.36). Patients without private insurance had increased odds of delayed surgery (OR 1.41, 95%CI 1.31–1.53); however, no significant difference was demonstrated for surgeries performed at private hospitals. A patient admitted to a low-volume hospital without private insurance was at 2.63 higher OR of delayed surgery compared to a privately insured patient presenting to a high-volume hospital (OR 2.63, 95%CI 1.53, 4.51).

**Table 3:** Factors affecting delayed surgery—multivariate multilevel logistic regression

	Adjusted OR (95% CI)	*P*
Patient characteristic		
Age (years)		
50–54	Ref	Ref
55–59	0.98 (0.89–1.09)	0.7373
60–64	1.13 (1.03–1.25)	0.0127
65–69	1.13 (1.02–1.25)	0.0151
70–74	1.46 (1.32–1.62)	< .0001
75–79	1.76 (1.57–1.97)	< .0001
80–84	2.14 (1.87–2.44)	< .0001
85 +	2.11 (1.84–2.42)	< .0001
Male sex	1.61 (1.52–1.69)	< .0001
CCI Score		
0	Ref	Ref
1	1.14 (1.06–1.23)	0.0008
2	1.36 (1.22–1.52)	< .0001
3 +	1.32 (1.19–1.47)	< .0001
Medicare-only insurance	1.41 (1.31–1.53)	< .0001
ICU admission	0.52 (0.45–0.62)	< .0001
Pancreatitis	1.86 (1.59–2.16)	< .0001
Cholangitis	2.69 (2.17–3.32)	< .0001
Hospital characteristic		
Hospital referral status		
Tertiary referral	Ref	Ref
Public non-tertiary	0.85 (0.58–1.23)	0.3878
Private	1.21 (0.80–1.82)	0.3667
Mean surgical volume		
High (157 to 266 per year)	Ref	Ref
Medium (53 to 156 per year)	1.73 (1.08–2.75)	0.0219
Low (0–52 per year)	2.02 (1.22–3.36)	0.0066

Model controlled for hospital of surgery—assigned as random effect in model

*OR* Odds ratio, *CI* confidence interval, *Ref* reference, *ICU* intensive care unit, *CCI* Charlson Comorbidity Index (0 = no comorbidity)

Admission to ICU at any time during index admission was also associated with lower odds of delayed surgery (OR 0.52, 95%CI 0.45–0.62). Neither private nor public non-tertiary hospital status was individually statistically significant.

## Discussion

This study presents the first population-level analysis of cholecystectomy for acute cholecystitis in an older Australian cohort. Factors identified as significantly associated with delayed surgical intervention were increasing age, male sex, increasing comorbidity the presence of additional biliary pathology, Medicare-only insurance, admission to a low- or medium-volume hospital and not needing ICU admission during the index hospitalisation.

While early laparoscopic cholecystectomy is recommended as the treatment of choice for acute cholecystitis, a recognised subgroup of individuals will inevitably be too unwell, unwilling or unable to undergo early surgery. Identifying this poorly defined subgroup is important given their typically worse outcomes and disproportionate burden on healthcare systems [3, 15, 16]. In adults of all ages, the observed proportion of early cholecystectomy for acute cholecystitis in the current literature ranges from 50 to 84% in cohorts from Australia, Japan and North America [8, 17, 18]. In the Australasian context, an early cholecystectomy rate of approximately 80% has been described by Poole et al. as an achievable benchmark for management of acute cholecystitis [16]. The rate observed in this cohort (83%) compares favourably to global rates, even in those aged over 85 years (*n* = 1517, 74%).

Factors affecting the ability to perform early cholecystectomy can be divided into patient factors or hospital/system-based factors [16]. Patient factors include advanced comorbidity, refusal of treatment and personal/social reasons. System-based factors include surgeons’ experience with advanced laparoscopic techniques, hospital service capacity—including emergency theatre time (seven days a week), radiology, ICU and interventional endoscopy services.

This study demonstrated a statistically significant increase in the risk of delayed surgery with age >65 years, male sex, comorbidity and concurrent biliary pathology. For some patients there may be the potential to optimise the medical management of comorbidities prior to surgical intervention and this should be determined through timely medical assessment so that only those whose risk profile can be altered are delayed. While advanced age is an established independent risk factor for poorer surgical outcomes, there is no established age limit at which cholecystectomy might be contraindicated. To that end, the concept of assessing frailty rather than using age as a determinant of surgical timing is becoming increasingly important [3].

Older, comorbid, rural males were over-represented in the delayed surgery groups. This could be explained by the higher frequency of antiplatelet or anticoagulant use in both males and older patients [19]. Our study could not specifically collect medication data, though the increased comorbidity is a surrogate indicator. Given the state of NSW’s large size, the rural population faces greater travel times and is also older compared to metropolitan areas (median 42.5 vs. 37.1 years) [20].

Admission to ICU and surgery in a high-volume hospital were associated with decreased odds of delayed surgery. Admission to ICU is a surrogate marker of disease severity and may suggest that presentations of severe acute cholecystitis are well supported by ICU services in NSW to facilitate early cholecystectomy. This may be via an intensive pre- and post-operative medical optimisation in ICU, or enhanced patient advocacy by ICU staff. As in our cohort, higher institutional volumes of cholecystectomy have been associated with better outcomes [21]. In order to safely achieve the highest rates of early surgery, advanced laparoscopic techniques and the associated ancillary services are required. Our study suggests hospitals performing more than three cholecystectomies for acute cholecystitis per week achieve higher rates of early surgery.

The association of Medicare-only insurance with delayed surgery suggests an inequality in access to timely surgery for patients without private health insurance. Australia has universal health insurance; however, approximately half the population choose additional private hospital insurance. All emergencies are initially referred to public hospitals, with private facilities presenting a subsequent transfer option for early surgery. There is little doubt that access to surgery through the private health system frees up capacity in the public system but investment in streamlined acute care pathways and timely access to theatres in the public system is also required. Given the significant increase in overall length of hospital stay with delayed surgery, the business case for prompt management for the vast majority of patients is compelling [22, 23].

For over a decade, many public hospitals in NSW and Australia have increasingly adopted dedicated acute surgical care services—often labelled “Acute Surgery Units” (ASU). There is ample evidence to support ASU models as cost-effective and safe, though their form and funding remain variable, particularly with regard to dedicated emergency theatre time [24–26]. This may represent an area for policymakers and administrators to expand services and possibly reduce inequalities in care.

Our data did not show any difference in mortality between the two groups. However, this study is not a prospective study of surgical decision-making, and we did not seek to differentiate those patients appropriately managed with delayed cholecystectomy. The observed 30-day mortality rates in the literature range from 0.5% in uncomplicated, young adults up to 4% in higher-risk patients [6, 27–30]. Given our cohort included older, comorbid patients, including those requiring ICU admission, our unadjusted mortality rates were comparatively low in all age groups.

This study’s strengths include a large, real-world cohort that includes the private sector and sophisticated methods of measuring and controlling for individual and institutional factors. Despite this, the study is limited by the nature of retrospective administrative data. While the models used in this study identified factors that are associated with delayed surgery and worse post-surgical outcomes, as a retrospective study this cannot be considered as causation. The potential for residual confounding from our inability to accurately determine severity of cholecystitis was partly mitigated by excluding those undergoing cholecystostomy and adjusting for those with comorbidity, concurrent biliary disease, or requiring ICU. Any variation in severity can be expected to be distributed evenly across the cohort after adjusting for demographics. Due to the limitations of data recorded in the APDC, there remains a lack of detail regarding other covariates of interest. On a patient-level ASA score, body weight and frailty score would be valuable though difficult to modify. At a hospital level, the availability of after-hours theatre time, presence of an acute surgical unit, surgeon experience and level of training, ERCP, radiology and ICU services are all relevant and potentially modifiable factors not assessable using our current database.

## Conclusion

A high proportion of older adults with cholecystitis are undergoing early cholecystectomy in NSW. Results of this study support the safety and efficacy of early cholecystectomy in older patients and highlight several modifiable factors which policymakers, hospital managers and care providers can address to further improve current models of care for acute cholecystitis. Delay to cholecystectomy, for acute cholecystitis, is associated with poorer outcomes in the population.
